# Extra-neural signals from severed nerves enable intrinsic hand movements in transhumeral amputations

**DOI:** 10.1038/s41598-022-13363-2

**Published:** 2022-06-17

**Authors:** Bahareh Ahkami, Enzo Mastinu, Eric J. Earley, Max Ortiz-Catalan

**Affiliations:** 1 Center for Bionics and Pain Research, Mölndal, Sweden; 2 Department of Electrical Engineering, Chalmers University of Technology, Gothenburg, Sweden; 3 Operational Area 3, Sahlgrenska University Hospital, Mölndal, Sweden; 4 Department of Orthopaedics, Institute of Clinical Sciences, Sahlgrenska Academy, University of Gothenburg, Gothenburg, Sweden

**Keywords:** Motor control, Neurology, Biomedical engineering

## Abstract

Robotic prostheses controlled by myoelectric signals can restore limited but important hand function in individuals with upper limb amputation. The lack of individual finger control highlights the yet insurmountable gap to fully replacing a biological hand. Implanted electrodes around severed nerves have been used to elicit sensations perceived as arising from the missing limb, but using such extra-neural electrodes to record motor signals that allow for the decoding of phantom movements has remained elusive. Here, we showed the feasibility of using signals from non-penetrating neural electrodes to decode intrinsic hand and finger movements in individuals with above-elbow amputations. We found that information recorded with extra-neural electrodes alone was enough to decode phantom hand and individual finger movements, and as expected, the addition of myoelectric signals reduced classification errors both in offline and in real-time decoding.

## Introduction

Motorized upper limb prosthetic devices are preferably controlled using myoelectric signals from muscles remnant to the amputation. Unfortunately, the more proximal the amputation, the fewer muscles are available to command the robotic joints needed to restore full function. A few ways to overcome the lack of independent and dedicated myoelectric control sites are to utilize machine learning algorithms^1^, to reconstruct the stump anatomy^2^, and a combination of both^3,4^.

Algorithms to decode motor volition have been explored since the 1960s^5^ and have further improved to include proportional^6^ and simultaneous control of a few degrees of freedom^7,8^. More recently, myoelectric pattern recognition technologies have become commercially available and spreading in clinical use. The use of this technology has been shown beneficial even in patients with surgical reconstruction^3^. Surgical reconstruction techniques such as Targeted Muscle Reinnervation (TMR) allow for new myoelectric sites to become available to surface electromyography (EMG) recordings^9^. TMR consists of transferring nerves severed by the amputation to remnant muscles that no longer actuate the missing joint, and thus can be surgically denervated to then host the transferred nerve. An alternative way to utilize muscles as biological amplifiers of neural signals is to dissect the severed nerve into its constituent fascicles and then use a free muscle graft to provide an innervation target, also known as Regenerative Peripheral Nerve Interfaces (RPNIs)^10^. However, the size of an RNPI is too small to be recorded from the surface of the skin creating the need for implanted electrodes^4^.

In addition to providing access to deeper or smaller muscles, implanted electrodes solve many of the issues related to the electrode–skin interface that plagues surface EMG recordings^11^. As early as 1982, De Luca et al*.* claimed the feasibility of recording neural signals from severed nerves in humans^12^, which was later confirmed in long-term amputations^13,14^. Intra-neural electrodes penetrating the blood-nerve barrier to access individual fascicles has since become the preferred method to record information within nerves in humans^15–22^. Direct neural recordings could be used to complement the information provided to decoding algorithms to improve reliability and increase the number of discriminable movements. However, no clinical implementation of this strategy has been performed, arguably because intra-neural electrodes have yet to prove years of long-term stability in peripheral nerves. Whereas extra-neural electrodes have shown long-term stability, this has been done in applications related to stimulation rather than recordings^23,24^.

In this study, we examine the feasibility of recording motor neural signals using non-penetrating neural electrodes and the viability of said signals for decoding phantom motor volition. The most common type of extra-neural electrodes are cuffs that wrap around the nerve. Cuff electrodes have shown to be long-term stable^23,24^ but mostly capture information from the peripheral fascicles, and thus it was important to verify if this could be a suitable neural interface for decoding motor volition of lost distal joints. In this study, we showed that it is possible to record enough neural information using cuff electrodes to allow for the discrimination of movements related to the missing hand, either alone or in combination with myoelectric signals from remnant muscles in transhumeral amputations (Video 1).

## Results

**Figure 1:**
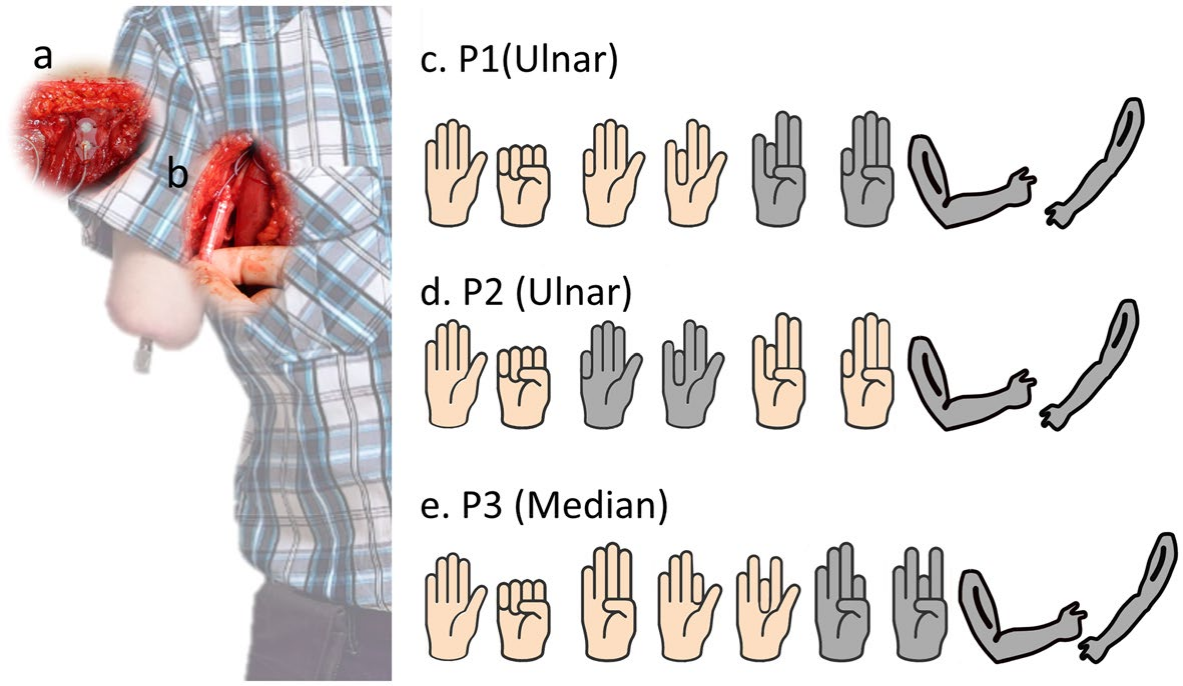
(**a**) Implanted muscular electrodes (biceps and triceps muscles), (**b**) extra-neural electrodes around the nerve (P1 and P2 Ulnar, P3 median), (**c–e**) hand gestures attempted by the subjects in their phantom hands. Grayed gestures only used in offline experiment.

In three participants with transhumeral amputations (P1-3), electroneurographic (ENG) signals were recorded using a cuff electrode implanted in the median or ulnar nerves severed by the amputation and that had no muscular targets. In addition, EMG signals were recorded using epimysial electrodes implanted in the biceps and triceps muscles (Fig. 1a,b). The cuff electrodes had a “mixed tripolar” contact configuration optimized for neural recordings^25^, in which three semi-independent channels were available. Access to the implanted electrodes from outside of the body was made possible via a permanent percutaneous neuromusculoskeletal interface^24,26^.

Production of motor action potentials through a desired fascicle can be a difficult task when the end effector is missing, and thus no feedback is available to verify the success of the motor command. In this study we used simple bar plot to train participants on the execution of motor commands using real-time biofeedback for 15 min prior to the recording session. Participants used a simple graphical user interface that showed the magnitude of each ENG signal in real-time to identify how motor commands could be executed to produce a discernible change signal strength (standard deviation). After this training session, participants were asked to execute four gross distal movements (hand open/close and elbow flexion/extension), and four or five finger movements related to the median or ulnar nerves (Fig. 1c–e).

**Figure 2:**
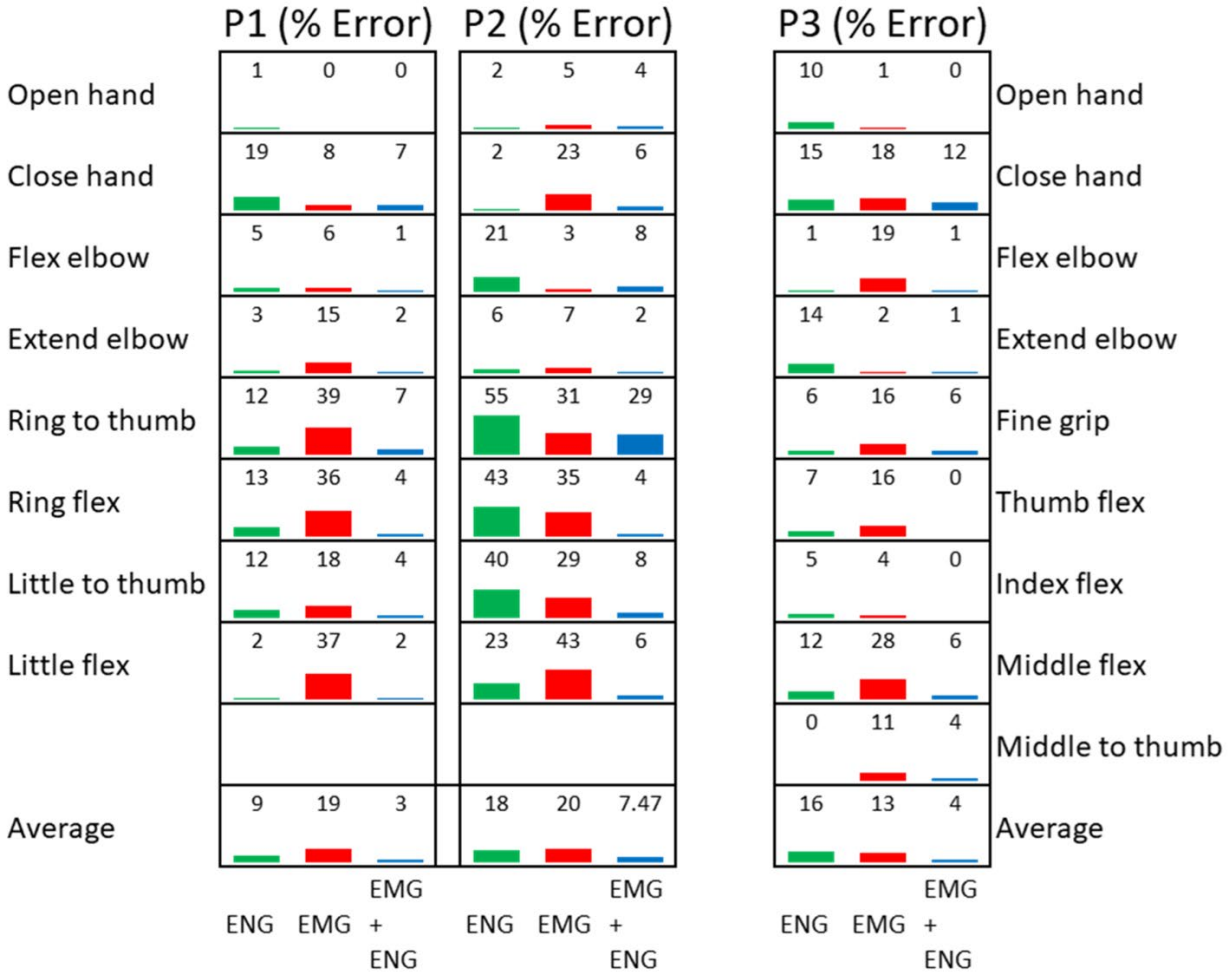
Offline decoding error on three participants (8 movements for P1–2 and 9 movements for P3) over the three different scenarios: (1) ENG alone, (2) EMG alone, and (3) EMG + ENG.

Offline classification using Linear Discriminant Analysis (LDA) showed that neural signals alone were enough to decode 8 movements with an error of 9% (± 4.7%) and 18% (± 17.9%) for P1 and P2, respectively. P3 decoding error rate on 9 movements was 16% (± 5.07%). Using solely EMG information resulted in errors of 19% (± 15%) and 20% (± 23.7%) for P1 and P2 and 13% (± 9.03%) for P3. As expected, discrimination errors decreased considerably when both ENG and EMG signals were used together, down to 3% (± 4%), 7.5% (± 14.4%), and 4% (± 3.8%) for P1, P2, and P3, respectively (Fig. 2).

**Figure 3:**
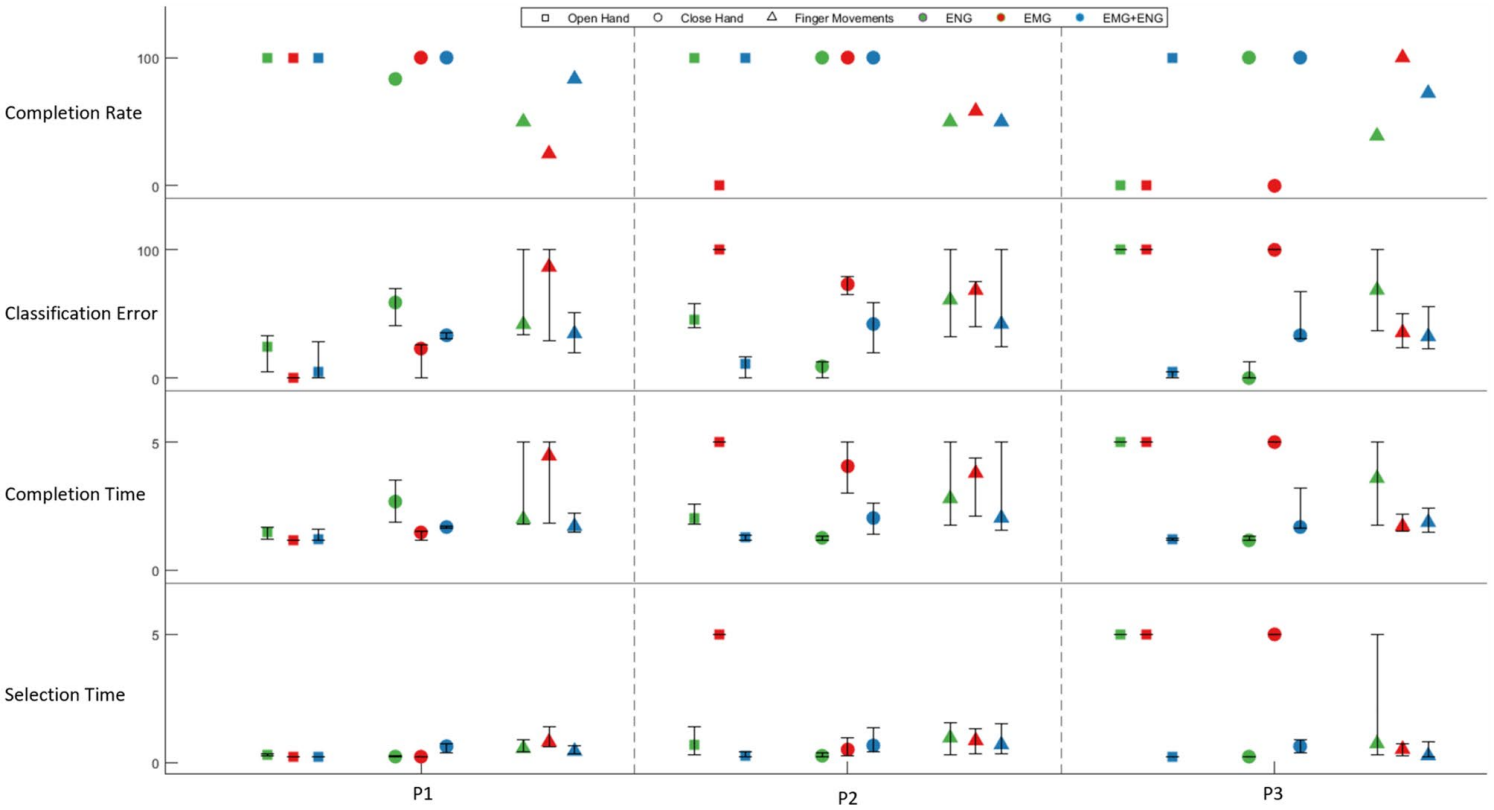
Online pattern recognition results from the Motion Test performed by the three participants (P1–3) over three different scenarios: (1) ENG alone (green), (2) EMG alone (red), and (3) the combination of EMG and ENG (blue). The outcomes are completion rate, median of classification error, median of reported completion time, and median of selection time. Finger movements include ring flex, little flex, for P1, ring to thumb, little to thumb for P2 and thumb flex, index flex, and middle flex for P3.

It is known that offline accuracy does not necessarily correlate with real-time discriminability^27^, therefore we conducted a real-time test in which the participants were asked to execute the different movements in random order. We employed the Motion Test^9^ and LDA algorithm as implemented in BioPatRec^28^. Motion Test was performed on a subset of the original movements. This subset was selected based on their Mahalanobis distance for a total of 4 (P1–2) and 5 (P3) movements. The real-time performance was observed in all participants, showing that that higher performance was achieved using ENG over EMG for P1 and P2, and marginal difference for P3 in most instances (Fig. 3). Like the offline results, the real-time performance improved when both ENG and EMG were used together (*p* < 0.01). The discrepancy between offline and real-time performance observed in the three participants was comparable to what is normally found in the literature (Fig. 3)^27,28^**.**

## Discussion

A major problem in artificial limb replacement is to effectively acquire and process neural signals to control artificial limbs. Here, extra-neural ENG data from three individuals with transhumeral amputation were used to demonstrate the feasibility of prosthetic control in both offline and online assessments. Our results indicate that neural signals can be recorded via cuff electrodes placed around nerves severed by an amputation (Fig. 1), and that these recordings contain enough information to decode intrinsic hand movements in above elbow amputations (Fig. 2). Although these findings seem to be unprecedented in literature and hold a promising venue for allowing the control of distal joints, they are only indicative for our particular and relatively small population of participants. A different placement of the cuff electrodes might change the content of information and consequently the individual control possibilities. The electrode configuration within the cuff and whether the nerves are innervating a muscle or terminated in a neuroma would also impact the quality of the extractable motor neural information. Similarly, the quality and length of the training that patients undergo to be able to produce usable motor signals is likely to have a considerable effect on the final outcome. For all these reasons, further work in a larger patient population is necessary to generalize our results.

Our online results indicate that the combination of EMG and ENG is not necessarily always the most optimal solution for all the movements. Instead, selective separation of information sources to their anatomically relevant joints, or a dynamic combination of the two bioelectric sources, might be preferable depending on the target movements and participants. For example, using ENG channels to feed information exclusively to decoders specialized in the relevant finger movements in a parallelized or cascaded structure of decoders.

**Figure 4:**
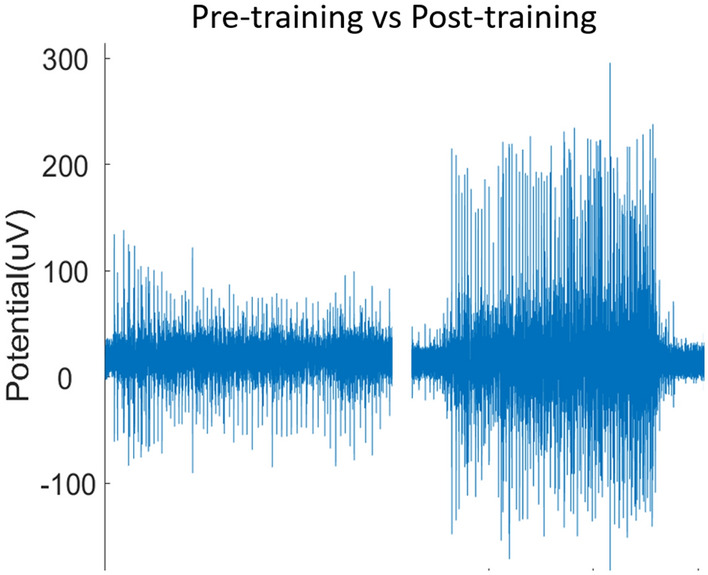
ENG signal from participant 1 during ring finger flexion. The plots represent the ENG signal before and after the training procedure used.

Previous literature has demonstrated the importance of receiving real-time biofeedback when training phantom limb movements^29,30^ . An interface, as simple as vertical bars displaying dynamically the level of activation per channel, can be beneficial when exploring phantom movements which are not normally attempted in the daily activities of people with upper limb amputation. Our participants (re)learned how to perform and differentiate movements with our simple training approach (Fig. 4 as example). However, investigating appropriate training routines is another area that requires further research. There are many other training methods that have showed promising results^29,31–34^, but our simple approach was enough to explore the main question in this study, which was the feasibility of decoding motor intent for extra-neural signals. Future work could focus on training the participants to produce distinct ENG signals despite of the functional relevance of the physiologically intended movement (*e.g.*, little finger flexion), and then use said ENG signals to control a prosthetic movement of more functional relevance (*e.g.*, phantom little finger flexion to prosthetic wrist rotation). Alternatively, participants could be training on non-physiological mapping right from the beginning.

This study also underlined the need for high-quality recording hardware and surgical implantation procedures that allow to attenuate crosstalk between neural and muscular signals. The low selectivity of extra-neural electrodes together with the impossibility to precisely place electrodes close to desired motor fibers presented serious challenges in this study. These limitations forced us to iterate over preliminary explorative sessions where, together with the participants, we sought for potentially controllable finger movements. This explains the variation of the target movements between participants, as well as an equivalent functional grasp in a real hand prosthesis. The small cohort of research participants is partially due to the involved technology; few people with transhumeral amputations have the implanted sensors required for this study. Ultimately, our purpose was to demonstrate the feasibility of utilizing extra-neural signal for decoding motor volition, and more pragmatically, to enrich the information content provided by EMG to enable intrinsic hand movements at the transhumeral amputation level.

It has been shown that the performance of many machine learning algorithms is practically the same in the decoding of hand movements using myoelectric signals^28,35,36^, and this is regardless of classifier’s capability to handle non-linearities, and the signal features employed^37^. Extra-neural recordings from cuff electrodes are often like EMG because they consist of compound action potentials rather than distinct single ones (see Fig. 4), and thus other approaches such as using firing rates are less suitable. Nevertheless, an area for future research in the decoding of extra-neural signals with non-linear classifiers and the use of more sophisticated approaches such as deep learning.

## Methods

### Participants

Three individuals with transhumeral amputation were enrolled in this study. Informed consent was obtained from all subjects prior to participation, in accordance with the provisions of the Declaration of Helsinki. All participants were implanted with the e-OPRA system (Integrum, Sweden) allowing for interfacing a prosthetic arm with the user’s bone, muscles, and nerves^24^. Epimysial electrodes were implanted in the Biceps Brachii and Triceps Brachii muscles, and an implanted cuff electrode was placed around the ulnar nerve for P1 and P2, and around the median nerve for P3. P1 was implanted in 2017, P2 was implanted in 2013, and P3 implanted in 2018. All participants have been using a myoelectric prosthesis from before and after implantation, however no participants have used ENG to control their prosthesis prior to this study.

This study was approved by the Swedish Regional Ethics Committee in Gothenburg (769-12).

### EMG and ENG recording system

A custom designed amplifier was used in this experiment. It consisted of five differential channels for the three contacts of each cuff electrodes (ENG) and the two epimysial electrodes (EMG). For the EMG channels, the gain was set to 500 V/V and the signal filtered with a 4th order Butterworth high-pass filter at 20 Hz and a 2nd order Butterworth low-pass filter at 1000 Hz. For the ENG channels, the gain was set to 20,000 V/V and the signal filtered with a 4th order Butterworth high-pass filter at 20 Hz and a 2nd order Butterworth low-pass filter at 4000 Hz. The amplified and filtered signal was then sampled at 8000 Hz and digitized at a 16-bit resolution using an analogue to digital conversion card (6212NI-DAQ, National Instruments, USA). The BioPatRec open-source platform was used for recording, decoding, and evaluation^28^.

### Target movements and recording sessions

All patients executed open hand, close hand, flex elbow, and extend elbow. On top of these movements, another set of finger movements was added to each participant according to the nerve in which the cuff electrode was implanted e.g., ulnar, or median nerve. The rationale was to select target movements related to fingers typically innervated by the implanted nerve. The participants with implanted cuff on the ulnar nerve (P1, P2) were assigned ring flex, little flex, little to thumb, and ring to thumb finger movements. The participant with a cuff electrode implanted on the median nerve (P3) was assigned thumb flex, index flex, middle flex, index to thumb, and middle to thumb finger movements. Each movement was repeated 5 times.

### Preprocessing and offline pattern recognition

We employed a well-known signal processing chain^38^, in which four common time-domain features (mean absolute value, slope changes, zero crossings and waveform length^39^) were extracted from overlapping time windows (200 ms with 50 ms increment). Eighty percent of the feature vectors were used in the training set, and 20% for the test set. The classifier was reiterated ten times and the highest classification accuracy was considered. To compare the ENG and EMG content of information, the offline classification accuracy was calculated considering (1) the ENG channels alone, (2) the EMG channels alone, and (3) the combination of EMG and ENG channels.

### Training software interface

A training interface based on biofeedback was used in this study with dual purpose: firstly, to get the participants accustomed to their particular set of target movements, and secondly, to incentivize movement distinction. This training software provided real-time biofeedback by displaying the standard deviation of each acquired channel independently via bar plots while the participant performed the movements in sequence. In this way, the software provided real-time feedback on the relevance of each channel to each movement, underlying visual patterns of muscular synergies and ultimately facilitating the learning and adaptation process.

### Online pattern recognition

The viability of pattern recognition was assessed in real-time using a well-known virtual assessment test, namely the Motion Test^9,28^. The Motion Test requires the participants to perform target movements randomly prompted on a screen. The target movements were selected as a subset of the offline movements.

This subset was defined individually for each participant by maximizing the Mahalanobis distance between the movements, omitting the movements with the most conflicts. The resulting subsets were Ring flex, Little flex for P1; Ring to thumb, Little to thumb for P2; and Thumb flex, Index flex, Middle flex for P3.

Twenty correct predictions had to be achieved in total within five seconds to consider a task completed, changed from the original test which required 10 correct predictions^28^. The output metrics of the Motion Test were:

- Completion rate: the percentage of requested movements that were completed within the given timeout (5 s).
- Completion time: the time needed to achieve 20 correct predictions.
- Selection time: the time needed to produce the first correct prediction.
- Online predicted error: the ratio of the number of incorrect predictions and the total number of predictions within the completion time.

Each target movement was repeated six times per Motion Test and the method of classification was LDA. Real-time test was performed considering (1) the ENG channels alone, (2) the EMG channels alone, and (3) the combination of EMG and ENG channels.

### Statistical analysis

Datasets were analysed using built-in statistics functions of MATLAB 2018b (MathWorks, USA). We quantified the effects of the participants, signal types, and movements on the outcome measures (classification error, selection time, completion time and completion rate). For this scope, a general linear model was defined with the following form:

$$Outcome\, measure\, \sim\, Movement\,+\,Signal\,+\,Patient$$

where *Movement* and *Signal* were categorical fixed factors, and *Patient* was a random factor. Then, pairwise comparisons were performed on the significant parameters of linear model to analyze differences between levels. Lastly, corrections for all comparisons were made with Holm-Bonferroni correction factors.

## Supplementary Information

Supplementary Video 1.

Supplementary Legends.
