# Supplementary material for: Extra-neural signals from severed nerves enable intrinsic hand movements in transhumeral amputations
Source: Sci Rep. 2022 Jun 17;12:10218. doi: 10.1038/s41598-022-13363-2 (PMC9206000; doi:10.1038/s41598-022-13363-2)
Supplement: Supplementary file 2 — Supplementary Legends. [file 41598_2022_13363_MOESM2_ESM.docx]

Supplementary Video Legends

Supplementary Video 1. Research Participant moving prosthetic hand using signals from nerve and muscle.

Video demonstrating one of the participants moving the prosthetic hand in real-time using neural and muscular signals. Movements are Open Hand, Close Hand, Ring flex and Little flex. These movements are related to the Ulnar nerve.
